# Transylvania University and the Navy

**Published:** 1845-07

**Authors:** 


### TRANSYLVANIA UNIVERSITY AND THE NAVY.

An article appeared in the *New York Herald* of the 17th of April last, purporting to be a letter from a Washington Correspondent, containing the following statement:

"It is a remarkable fact, mind that, a remarkable fact, that at the last examination of applicants for the appointment of Assistant Surgeons for the Navy, out of thirty examined, only sixteen were found qualified, and that the fourteen rejected as incompetent, were graduates of the Medical College of Lexington, Ky., and Cincinnati, Ohio. Pray, Drs. Dudley and Locke, how is this? Is it possible your Professors award diplomas initialized M. D., to green-horns, not even qualified for Assistant Surgeons in the Navy? Look sharp, gentlemen, look sharp, or the Louisville College will carry off the premium."

Immediately on the appearance of this slanderous charge against these respectable institutions, one of the Transylvania professors addressed a letter on the subject to Dr. Barrington, a member of the Navy Board, and received the following reply, which we have much pleasure in copying.

PHILADELPHIA, April 21st, 1845.

SIR:—I have this day received your letter of the 15th inst., proposing the following queries, viz:

"How many candidates for examination for the place of Surgeon or Assistant Surgeon in the Navy, have you known to have reported themselves as graduates of Transylvania University!"

"How many of the same have been found unqualified?"

It gives me pleasure to state, in reply, that of the candidates for admission into the Medical Department of the Navy, rejected by the last Board of Naval Surgeons, not one was a graduate of either Lexington or Cincinnati. Nor has any graduate of the Transylvania University yet presented himself before any Board of which I have been a member.

I am, very respectfully,
Yours, &c.
SAMUEL BARRINGTON,
*Surgeon U. S. Navy.*

To THOS. D. MITCHELL, Prof. Mat. Med. and Therap. Transylvania University.

